# Multiple long-term conditions as the next transition in the global diabetes epidemic

**DOI:** 10.1038/s43856-025-00742-9

**Published:** 2025-02-14

**Authors:** Edward W. Gregg, Naomi Holman, Marisa Sophiea, Shivani Misra, Jonathan Pearson-Stuttard, Jonathan Valabhji, Kamlesh Khunti

**Affiliations:** 1 School of Population Health, RCSI University of Medicine and Health Sciences, Dublin, Ireland; 2 School of Public Health, Imperial College London, London, UK; 3 NHS England, Wellington House, London, UK; 4 Department of Diabetes and Endocrinology, St Mary’s Hospital, Imperial College Healthcare NHS Trust, London, UK; 5 Division of Metabolism, Digestion & Reproduction, Faculty of Medicine, Imperial College London, London, UK; 6 Lane Clark & Peacock LLP, London, UK; 7 Chelsea & Westminster Hospital NHS Foundation Trust, London, UK; 8 Diabetes Research Centre, University of Leicester, Leicester, UK

**Keywords:** Endocrine system and metabolic diseases, Health care, Endocrine system and metabolic diseases

## Abstract

Several transitions, or new patterns and dynamics in the contributors and health outcomes, have altered the character and burden of the multi-decade, worldwide growth in prevalence of type 2 diabetes (T2DM). These changes have led to different needs for prevention and care. These dynamics have been driven by diverse demographic, socio-economic, behavioural, and health system response factors. In this Perspective, we describe these transitions and how their attributes have set the stage for multimorbidity, or multiple long-term conditions (MLTCs), to be the next major challenge in the diabetes epidemic. We also describe how the timing and character of these stages differ in high-, middle-, and low-income countries. These challenges call for innovation and a stronger focus on MLTCs across the spectrum of cause, effectiveness, and implementation studies to guide prevention and treatment priorities.

## Abstract (web summary)

Gregg et al. discuss the long-term changes seen in the global diabetes epidemic, including increasing survival, early-onset type 2 diabetes, and sub-optimal prevention. These changes have set the stage for multimorbidity, or multiple long-term conditions, to be the next major challenge for type 2 diabetes.

## Introduction

The global pandemic of type 2 diabetes (T2DM) is often viewed as a long-term by-product of the great epidemiologic transition, wherein in the previous century, human patterns of mortality and disease exchanged high rates of mortality due to infections and maternal and child mortality for lower mortality rates driven by chronic conditions such as cardiovascular disease (CVD), cancer, and T2DM^1,2^. However, there have been differing changes in incidence for specific chronic conditions. For example, deaths due to CVD decreased beginning in the 1970s and 1980s whereas T2DM, lacking effective policy or health care options for prevention, accelerated in the 1990s^2–5^. Although recent signs of a decrease in T2DM incidence in some countries have emerged^6,7^, most countries of the world continue to experience unrelenting growth of the problem^6–8^.

The T2DM pandemic has itself been complex and multi-phasic. Underlying the overall increases in prevalence, have been several transitions across distinct periods of the T2DM epidemic that impacted the burden and approaches to care, prevention, and research^9–12^. In this Perspective, we explore how these transitions have set the stage for multiple long-term conditions (MLTC, or multimorbidity) impacting morbidity, health services, and approaches to prevent T2DM.

### Seeding and steady growth of the problem

**Fig. 1:**
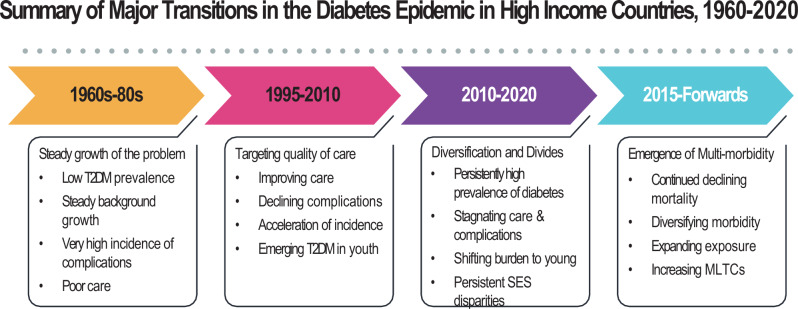
Summary of major transitions in the global diabetes epidemic, as experienced by high income countries, 1960–2020. The figure describes key patterns in prevalence, incidnece, complications, care, and socio-demographic factors affecting the T2DM pandemic. T2DM: Type 2 diabetes mellitus; DM: diabetes mellitus; SES: socio-economic status; MLTC: multiple long-term conditions

The first phase of growth in the T2DM epidemic, evident in the 1970s and 1980s, consisted of steady, gradual, increases in prevalence in high income countries (HICs) and more rapid increases in selected low- and middle-income countries (LMICs) undergoing rapid urbanization, nutritional and other sociocultural changes associated with westernization^5,13–15^ (Fig. 1). This was associated with a global nutrition transition, with dietary shifts toward refined carbohydrates, added sweeteners and edible oils, and away from legumes and fresh fruits and vegetables. Declining physical activity levels contributed further and collectively they are cited as driving an environment conducive to increases in obesity and T2DM^16,17^. However, where data were available, diagnosed diabetes prevalence rates were quite low, at around 2–4% in the US, UK, and Northern Europe^5,18,19^ compared to recent global estimates, which have surpassed 10% of adults^5,8^. Further, this low diabetes prevalence contrasted with very high rates of severe complications, including a 20-fold increased rate of lower extremity amputation, 10 times the rate of end stage renal disease (ESRD), and three times the risk of CVD^20–26^. These high complication risks were attributed to suboptimal diabetes care, risk factors and self-management practices. For example, estimates from the some HICs in the mid-1990s showed that large segments of the population with diagnosed diabetes had HbA1c levels well over 9% (75 mmol/mol) and blood pressure levels > 140/80, levels considered to be a sign of inadequate management by current standards^27–29^. Thus, perception of the problem centred more on the complications accompanying T2DM rather than the incidence and potential prevention of T2DM.

### Targeting quality of care

The juxtaposition of low diabetes prevalence but high incidence of complications among people with diabetes, combined with new evidence for the preventability of diabetes complications with glycemic, blood pressure, and multifactorial CVD risk factor management for both type 1 diabetes and T2DM, spurred a second, distinct period between 1995–2010^30–32^. A focus on improving quality of care became the primary public health response, including concerted attention to improve the structure and organization of health services for chronic disease care^9,28,29,33^ (Fig. 1). Collectively, these improvements in care delivery are credited with leading to broad reductions in rates of incidence of myocardial infarction, stroke, lower extremity amputations, and ESRD during the 2000s and contributing to a step change in the quality and length of life for people with the condition in selected high income countries^21,34–37^. However, these advances did not bring care close to optimal levels, as only a minority of people with diabetes met guideline-directed target levels of risk factor control and preventive care practices^38^. Further, in the background of these improvements, increases in diagnosed T2DM incidence accelerated,^4,5,7,8^ accompanied by a dramatic growth in obesity and T2DM in youth and young adulthood, disproportionately affecting sub-populations of lower socio-economic and non-white populations in the US, UK, and Europe that continue today^11,39,40^.

### Stagnation, diversification, and divides

Following the encouraging improvements in diabetes care and reduction in complications, the period from 2010 to the present reveals new, unanticipated dynamics. First, the disproportionate decrease in CVD mortality of the prior decades was accompanied by diversification and relative increases in other forms of morbidity^12,41^. (Fig. 1). For example, among the populations with diabetes in the US and UK, hospitalizations and deaths due to vascular causes declined precipitously while other common causes, including kidney disease, respiratory infections and deaths due to cancers, liver disease and dementia have increased^37,42^. A similar analysis of hospitalizations in Australia among adults with diabetes shows that admissions due to severe stress and adjustment disorders, iron deficiency anaemia, kidney stones, and gastroenteritis have increased^43^. These studies are a reminder that diabetes is associated with many other conditions not traditionally regarded as complications of diabetes. Although less specific, and with weaker magnitudes of association than classic complications, the large number of conditions alongside their relatively high frequency in older adults, means they can have an important overall impact on diabetes-associated morbidity.

A second, important observation from this period has been an apparent stagnation of improvements in care and reductions in complications^44–46^. In the US, this has included a plateau in levels of glycemic, blood pressure, and lipid control and a resurgence in complications in adults with diagnosed diabetes and an increase in mortality rates in the general population of middle-aged white men. Increases in hospitalizations for acute hyperglycaemia and lower extremity amputations have been reported in the US, UK, and Australia, although there remains considerable international variation in these trends^47–51^. More broadly, there has been a slowing of improvements in CVD mortality in many countries, with notable effects on overall life expectancy in middle age men in the US^44^. In LMICs, trends in care and risk factor management are unclear but have been shown to be at a generally suboptimal level and worse than in HICs^52^. Further, drivers of a slowdown in improvements are also unclear but socio-economic recession and inequalities, as well as the challenges of heterogenous, early onset disease, have each been cited as possible factors^53,54^.

A third, unexpected trend has been an apparent peak and decrease in diabetes incidence itself in some settings. This has been paired with a plateau in prevalence in many HICs, although there is little evidence that this reflects broader global trends^6,45,47^ The decrease in incidence seen in high-income countries remains unexplained^6,7^. Some countries, including the US, UK, and Finland have invested in structured community-based prevention programmes. While these programmes have promising potential to reduce incidence in high-risk participants, their reach across the population needs to be substantial, such as that seen in England, to alter trends in incidence^55^. It is also possible that an accompanied emphasis on screening and testing for pre-diabetes, health promotion efforts and increased education and awareness of the scale of the diabetes threat, is helping turn the tide on the underlying risk behaviours. However, there has been limited data on trends in risk factors to confirm this. It is also possible that shifts in diagnostic criteria to HbA1c from fasting plasma glucose, or other changes in testing, have affected incidence of diagnosis^56^.

### Increased burden in the young

Many elements of the transitions described above were influenced or exacerbated by an age divide, cutting across aetiology, care, prevention and complications^57,58^. For example, the greatest relative increase in incidence and prevalence has been among the young. Among cases, the risk profiles at diagnosis and the excess risk of morbidity and mortality is greater in young than older populations compared to same-aged counterparts without diabetes^59–63^. Earlier age-at-onset of T2DM is associated with higher proportions of obesity, a more adverse behavioural risk profile, proportionately more people from racial/ethnic minorities, and worse risk factor control than those with later-onset T2DM and worse pregnancy outcomes compared to women with type 1 diabetes^59–62,64,65^. Where improvements have occurred in care, prevention, and outcomes, they have been driven by the older population, leading to a reduction in the age distribution of the population with diabetes-related complications^21^. The factors driving this shifting burden to the young are unclear, but socio-economic inequalities and a disproportionate vulnerability of high risk youth to obesogenic environments may play a large role^6,47,53,57,58^.

### The global high-to-low income divide

The transitions described above are mainly evident in HICs, as a large data gap in complications and morbidity among populations with diabetes leaves the status of LMICs unclear^36^. Where data exists, LMICs appear to have followed similar paths^5,66,67^. However, periods of acceleration of increases in diabetes incidence and prevalence were accompanied by even greater magnitudes and longer periods of increases in South Asia, the Middle East, North Africa, and Oceania^8^. In China, increases in burden have generally paralleled those of the US, but given the population size, disproportionately affected the overall global burden^68^. The greater growth in diabetes prevalence in LMICs is consistent with previously described nutritional and obesity transitions, as well as economic and commercial influences affecting the availability and affordability of unhealthy compared to healthy foods, and environmental risks affecting levels of physical activity^16,17,69^. These changes have also included a shift from persons of high socio-economic status (SES) to low SES having highest risk even in countries with histories of undernutrition alongside the long-term migration of people from rural to urban settings^70–73^. Despite the long-term role of urbanization, recent evidence also suggests that the current growth in obesity prevalence is being influenced more by increases in rural than urban settings^73^. Populations with diabetes in LMICs also have lower levels of preventive care services and risk factor management and higher relative risk of complications and mortality^67,72,74,75^.

The reductions in overall mortality rates across LMICs should be expected to benefit adults with diagnosed diabetes, but the lack of continuous diabetes surveillance systems with the ability to track denominators with diagnosed diabetes has limited published reports on causes-specific mortality or complications among populations with diabetes^76^. Thus, whether the long-term reductions selected complications and the apparent diversification of outcomes seen in HICs are also occurring in LMICs is unknown. Reports from India and Mexico showing considerably higher relative risks of mortality, CVD, and kidney disease than that shown in HICs raises the question of whether population trends and dynamics could also be different^67,77,78^.

### The emergence of MLTCs as the next diabetes epidemiologic transition

**Fig. 2:**
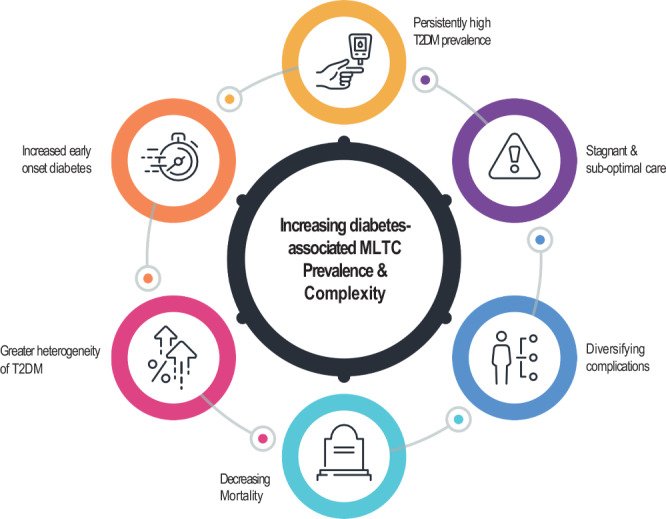
Primary drivers of the population-level increase in MLTC prevalence associated with type 2 diabetes. The figure describes key factors affecting population-level rates and trends in MLTCs. T2DM: Type 2 diabetes mellitus; MLTC: multiple long-term conditions

We have described a succession of major transitions in the diabetes epidemic, from seeding and growth of the problem to successes in care and prevention of complications with an underlying shifting burden to the young to an era of stagnating care, diversification of outcomes, and persistent socio-economic disparities. These transitions, accompanied by the co-factors of decreasing CVD mortality, early onset and greater heterogeneity of T2DM have set the stage for the next transition of the evolving diabetes epidemic in HICs, with MLTCs representing the next major challenge (Fig. 2).

Multimorbidity, or MLTCs, has been defined as the co-occurrence of two or more chronic conditions within a single individual, including non-communicable diseases of long duration like CVD or cancer, a mental health condition and mood disorders, and infections of long duration, such as HIV or hepatitis C^79^. The UK National Institute for Health and Care Excellence (NICE), definition also includes alcohol or substance abuse, symptom complexes such as frailty or chronic pain, sensory impairments and ongoing conditions that affect function, such as learning disability^80^. MLTCs may be further classified as concordant if they share a pathophysiological pathway with the index condition (e.g. diabetes and chronic kidney disease), or discordant if no obvious shared pathway exists, (e.g. T2DM and asthma), and complex if three or more conditions affect multiple bodily systems. Although the concept of concurrent conditions has historically been intrinsic T2DM, leading many to report concordance and cross-prediction of microvascular and macrovascular complications, public health agencies in the US, UK, as well as the WHO have prioritised the problem of MLTCs for chronic diseases as a whole^81–87^.

**Fig. 3:**
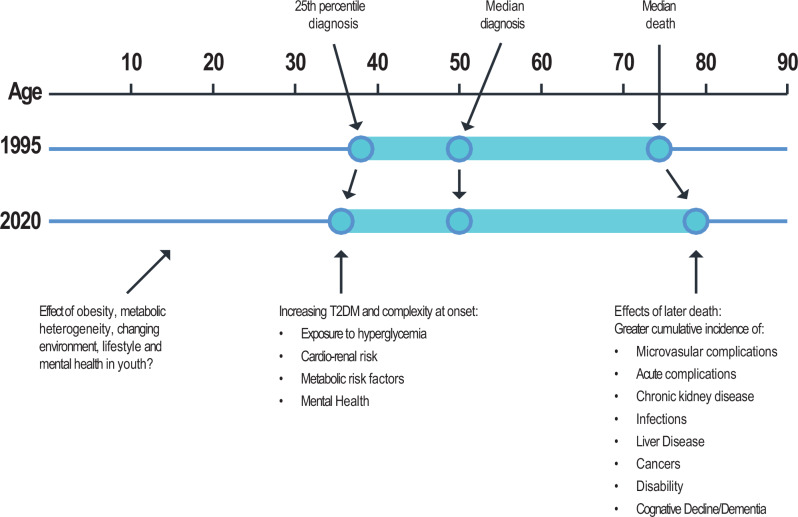
The relationship of age-dependent risk and the development of multiple long-term conditions associated with type 2 diabetes. Figure is hypothetical and not drawn from a specific population or data set. However, the estimates of median and 25th percentiles in the distribution of age at diagnosis are based on data from the US National Diabetes Surveillance System^108^. The estimates of change in average age of death are based on published modelled estimates that are also based on US national data^24^. T2DM: Type 2 Diabetes Mellitus.

The transitions in the diabetes epidemic could increase MLTCs by acting at both ends of the age distribution. In older adults, gains in longevity and diversifying complications will likely drive a persistent and higher prevalence of MLTC and cumulative morbidity even if incidence rates of complications decline (Fig. 3). In younger populations, the heterogeneous and adverse risk profile at diagnosis of obesity, and early onset comorbid conditions, particularly in ethnic minority populations, results in an earlier and longer exposure to adverse metabolic risk with potentially long-term effects on health service use, and work productivity during the life stage when optimal work productivity is expected^88^. The net effect of obesity, metabolic heterogeneity, and the changing environment on lifestyle and mental health before onset could also change the initial context. The lack of available new systems, structures, guidelines, or therapeutic approaches to reduce and effectively manage MLTCs or mitigate the influential effect of wider social determinants of health on MLTCs will create greater challenges.

The study of MLTCs has been limited by the complexity of their measurement and the heterogeneity of conditions, which has often relied on crude metrics^89^. Although a diverse taxonomy of MLTC metrics exists, quantified by diagnostic categories, drug use, physiologic measures^90^, and several weighted summary scores, the most commonly used approach is to sum the number of conditions from a pre-selected list of diagnosis codes. Although easily interpretable, this is also a blunt way of estimating the variation, severity, and impact of MLTCs. More recently, clustering approaches, including machine learning (ML)-based statistical methods, have attempted to identify distinct clusters or phenotypes. To date, these studies have studied distinct phenotypes based on clinical characteristics and health status at diagnosis, to predict risk for diabetes complications^91^. At least one study has shown that ML-derived clusters, in turn, are highly predictive of subsequent outcomes, possibly serving as a precursor for their incorporation into treatment models^92^. Distinct clusters of risk factors may also act earlier, such as in a pre-diabetes phase, or later, as MLTCs increase. A rapidly growing literature is attempting to understand how clusters of MLTCs develop longitudinally but the complexity involved has limited both interpretation and clinical utility to date^93,94^. Advances in identifying the underlying mechanisms and molecular drivers are likely to influence characterisation of prominent MLTC phenotypes in the near future^95^.

### The epidemiology and characteristics of diabetes and multimorbidity

The lack of standardized MLTC metrics leads to highly disparate prevalence estimates across studies^96^. One systematic review of 193 studies estimated the pooled prevalence of MLTC at 42.4%, with the prevalence being higher in older adults and in studies that included large numbers of conditions. Barnett et al. showed that the proportion with at least three conditions doubles roughly every decade of life, from about 5% at age 40 to more than 10%, 20%, and 40% at age 50, 60, and 70 years respectively^97^. Further, people in the lowest decile of social deprivation have a prevalence of three or more conditions 10 to 15 years earlier than those living in more affluent areas. Studies in the UK suggest that the prevalence of having three or four or more MLTCs has increased over time^98^, although the degree to which shifts in screening, diagnoses, or coding have affected trends over time is not clear.

Diabetes has consistently been shown to be one of the central components of MLTCs. Population-based studies have shown that at diagnosis of T2DM, 26% of men and 34% of women already have two additional conditions while 10–15% have at least three conditions^99^. In UK National Health System data, by age 50, one-third of persons with diabetes have at least three conditions, spend >20 years with them and die 11 years earlier than the general population^100^. The most common contributing conditions to MLTC in people with T2DM are hypertension, depression, coronary heart disease, chronic kidney disease, anxiety, arthritis, and atrial fibrillation. Analyses of clustering have suggested that MLTCs may follow particular, distinct patterns, including classic cardio-metabolic precursors (e.g. obesity, hypertension), later stage vascular conditions (atrial fibrillation, stroke, peripheral vascular disease), mental health conditions with a young onset, and mental health conditions clustered with chronic obstructive pulmonary disease and asthma^99,101,102^. The patterns of prominent comorbid conditions also vary considerably by age. Among older adults, classic complications like myocardial infarction, stroke, heart failure, and peripheral vascular disease play major roles, whereas in young adulthood mental health conditions and asthma also play large roles^100^, Although these increases are thought to be driven by an actual increase in burden of disease due to persistent prevalence and declining competing risk of cardiovascular disease mortality, the impact of changing diagnostic and coding practices and the assembly of more data sources have not been clarified.

### Aetiologic drivers and prospects for prevention and management

The aetiology of MLTCs in persons with diabetes stems from numerous factors and pathways. Studies in the general population have shown that age, socio-economic status (SSE), material deprivation (an inability to afford basic resources), obesity, physical inactivity, and smoking, are all important predictors of MLTCs^89^. The cardinal drivers of diabetes complications, which are hyperglycaemia, insulin resistance, hypertension, dyslipidemia, and inflammation, may affect non-traditional conditions contributing to MLTCs, such as liver disease, respiratory disorders, infections, cancers, mental health, and musculoskeletal problems^103^. Similarly, the toxic effects of excess glucose, dysfunction of arterial walls, and chromosomal variation affecting the aging process, may also play a role in MLTC development. A UK study showed that obesity dramatically increases risk of increased severity of MLTCs^104^. It remains unclear, though, whether the risk factors are fundamentally different from those of the constituent components or whether MLTCs represent incremental levels of severity along the same aetiological continuum.

It is important to understand what these transitions mean for the future of research and public health programmes. The rapid developments in precision medicine, multi-omics, artificial intelligence, and data science will each play a role in clarifying the diabetes-associated pathways and unique modifiable risk factors for prevention and management of MLTCs. However, if MLTCs are the next major transition in the diabetes epidemic, and the key drivers lie at both ends of the age spectrum as we suggest, the most practical priorities will lie in prevention, ongoing or routine care models and coordination, and globally, population monitoring. The key questions for prevention and management are whether the approaches (e.g., lifestyle interventions, self-management, better risk stratification, pharmaceutical interventions; better-integrated care) are distinct from what works for its constituent component or whether distinct, bespoke approaches are needed for MLTCs. The search for promising prevention approaches has centred around lifestyle interventions with physical activity to prevent functional decline and the use of multi-disciplinary teams to target the pharmacological, medical and self-management challenges of MLTCs, but consensus on the degree of effectiveness is still lacking^105^. Observational studies have suggested that weight loss in obese people can have beneficial effects in some concordant and discordant conditions but few weight loss intervention studies have evaluated the impact on MLTCs^106,107^. Better population monitoring, and with it, the need for more efficient and textured metrics, alongside simple measurements that can be applied internationally, are needed. There is also a need to expand the prevention agenda beyond HICs while improving the ability to monitor the problem using new metrics.

### Concluding remarks

In this Perspective, we have described several major transitions within the global T2DM pandemic that have shaped its long-term impact and now set the stage for the coming challenges. Underlying the long-term growth of global T2DM prevalence, declining CVD mortality, a period of improvements in care and outcomes, a diversification of complications in older adults, potential stagnation in long-term care improvements, and a shifting burden from older adults to middle-aged adults have all shaped a changing profile of type 2 diabetes morbidity. Collectively, these dynamics have set the stage for a high prevalence of MLTCs being an extensive and persistent challenge for care and prevention in the future. Whether LMICs follow the patterns seen in HIC will have enormous implications for the global burden of diabetes because most of the world’s individuals with diabetes reside in LMICs. It will be essential for future research in health services research, epidemiology, and implementation research to innovate to facilitate an effective population health response to the MLTC challenge.
